# Distinct clinicopathological features and treatment differences in breast cancer patients of young age

**DOI:** 10.1038/s41598-025-90053-9

**Published:** 2025-02-15

**Authors:** Rasmus O. C. Humlevik, Amalie A. Svanøe, Turid Aas, Anette Heie, Anna K. M. Sæle, Lars A. Akslen, Elisabeth Wik, Erling A. Hoivik

**Affiliations:** 1 Centre for Cancer Biomarkers CCBIO, Department of Clinical Medicine, University of, Bergen, Haukeland University Hospital, N-5021 Bergen, Norway; 2 Department of Pathology, Haukeland University Hospital, Bergen, Norway; 3 Department of Surgery, Haukeland University Hospital, Bergen, Norway

**Keywords:** Breast Cancer, Young Age, Stemness, Treatment, Survival, Breast cancer, Biomarkers, Breast cancer

## Abstract

The incidence of breast cancer in young women (aged under 40) is on the rise and is associated with more aggressive tumor characteristics and lower survival rates. Breast cancer is most frequently diagnosed in the sixth decade, and most research presents results based on data from older patients. By using large-scale clinico-pathologic and transcriptomic data from the Molecular Taxonomy of Breast Cancer International Consortium (METABRIC) (n = 1932), we aimed to explore age-related differences in treatment, tumor characteristics, and gene expression signatures. Young patients presented more aggressive clinico-pathologic features such as higher histological grade, more frequent lymph node metastasis involvement, and estrogen receptor negativity. Accordingly, age below 40 years was associated with lower mRNA expression of the estrogen- and progesterone receptors, encoded by *ESR1* and *PGR*, a higher proportion of the basal-like subtype, and increased transcription patterns reflecting stemness. Young breast cancer patients showed reduced survival, also within the basal-like subtype. We observed age-related differences in treatment, with more patients receiving chemotherapy among the young. Our results confirm a more challenging disease in young patients with breast cancer despite the more abundant use of chemotherapy. This argues for increased attention to young patients in current management and future research in breast cancer.

## Introduction

Despite the tight connection between cancer and aging, about 7% of breast cancer patients in higher Human Development Indexed countries are below 40 years at the time of diagnosis^1^. Moreover, the incidence among young breast cancer patients is on the rise^2^. Interestingly, breast cancer in young women is characterized by shorter overall survival and unique age-related challenges compared to older^3^. Young age is found to be an independent risk factor of poor prognosis even with updated treatment modalities, as described in a recent study from Canada^4^. The reason for this unfavorable outcome remains unexplained, and young women are still under-represented in research assessing risk-stratification models and clinical trials^5^. As a consequence, young women may face an increased risk of receiving unnecessary chemotherapy, potentially leading to overtreatment^6^.

It is well established that young women with breast cancer have higher rates of aggressive tumor features, such as larger tumors, higher tumor grade, lymph node involvement, hormone receptor negativity, HER2 receptor positivity, higher Ki67 expression and more aggressive molecular subtypes such as triple negative and HER2 positive^7^. The significance of these clinico-pathologic features in young patients is not well investigated. However, in a recent study from our group using in-house data, we found no prognostic value of Ki67 in patients below 40 years at time of diagnosis^8^.

Presence of cancer stem cells, found particularly associated with the basal-like subtype of breast cancers^9^, might contribute to the aggressive nature of these tumors. Stem-like properties of breast cancer is shown to cause resistance to chemotherapy and contribute to tumor progression^10^. Given the higher proportion of triple negative- and basal like subtypes among young patients, investigating age-related differences in cancer stemness features could offer insights into the unfavorable outcomes in this patient group. Only one previous study has suggested increased stem-like processes among the younger breast cancer patients^11^, but overall, this is a poorly understood topic for this patient group.

Here, we have studied the impact of age on breast cancer characteristics, survival, and treatment, by analyzing publicly available mRNA expression profiles and clinical data from the METABRIC cohorts.

## Methods

### Cohorts

We employed two datasets from The Molecular Taxonomy of Breast Cancer International Consortium (METABRIC) (invasive breast cancer; discovery and validation cohorts; n = 997 and n = 995). The two cohorts were comparable in terms of clinical- and demographic characteristics, but with notable differences that the validation cohort consisted of a higher number of patients with the Basal-like subtype, and that cases with low cellularity was included. The details of these cohorts are described in the original paper, Supplementary Table S1^12^. The METABRIC mRNA expression datasets were originally derived from fresh-frozen primary breast cancer specimens in Canada and UK, using the Illumina Human v3 microarray^12,13^. The patients in the METABRIC study^13^were enrolled between 1977–2005, at a time before the introduction of trastuzumab^12^. Clinico-pathologic data paired with mRNA expression data was downloaded June 2016 from the European Genome-phenome Archive (EGA). The median follow-up time for survivors were 117 months (range 1–303 months). The molecular subtypes were defined by the PAM50 classification^14^. Normal-like subtypes were excluded from the analyses.

### Gene expression signatures

The mRNA expression data was obtained as Log2 values and collapsed according to max-probe approach when multiple microarray probes covering the same gene were present^15^. The luminal progenitor and mature luminal signatures presented by Lim et al.^16^and two signatures reflecting stemness features^17,18^ were mapped to the METABRIC data. The cancer stemness score were calculated by a sum of expression values of signature genes. The Nestin score and luminal signatures were calculated by subtracting the sum of expression for the down-regulated genes from the sum of expression for up-regulated genes.

### Ethics statement

We confirm that all methods were carried out in accordance with relevant guidelines and regulations. Ethical approval was obtained from the University of Cambridge and the British Columbia Cancer Research Centre and written informed consent were obtained from the participants in relation to the original publication^12^. Approval to use the METABRIC data in relation to our study was received by the METABRIC Data Access Committee (2015). The results here are in part based upon data generated by the METABRIC study^12^. No additional approval was acquired as the study is based on publicly available data.

### Statistics

All statistical tests were analyzed using SPSS for Windows version 29 (IBM Corp, Armonk, NY). Two-sided p-value of 0.05 was considered significant. Associations between categorical variables were assessed using Pearson’s Chi-Squared test. Mann–Whitney U and Kruskal–Wallis tests were used to compare continuous variables across groups. Kaplan–Meier methods, with log-rank test to compare different categories, were used for survival analyses. Entry date was time of diagnosis and breast cancer specific survival was the endpoint. Patients who died from other causes than cancer were censored at the date of death. Multiple logistic regression was used to compare use of chemotherapy, hormone therapy, and radiation therapy between patients younger and older than 40 years at time of diagnosis.

## Results

### Young breast cancer patients have more aggressive tumor features

Following up on our previous study^8^, and aiming to explore the distribution of clinico-pathologic variables across age groups in independent cohorts, we separated the METABRIC cohort into three age groups. The youngest age group comprised of women below 40 years of age at time of diagnosis (n = 120, 6%, mean age 35, median age 36). The remaining patients were split into the age groups 40–49 (n = 306, 15.4%, mean age 45, median age 45) and ≥ 50 (n = 1566, 78.6%, mean age 66, median age 65) at time of diagnosis. Across the full dataset, mean and median age was 61 years (range 21–96 years old).

**Fig. 1:**
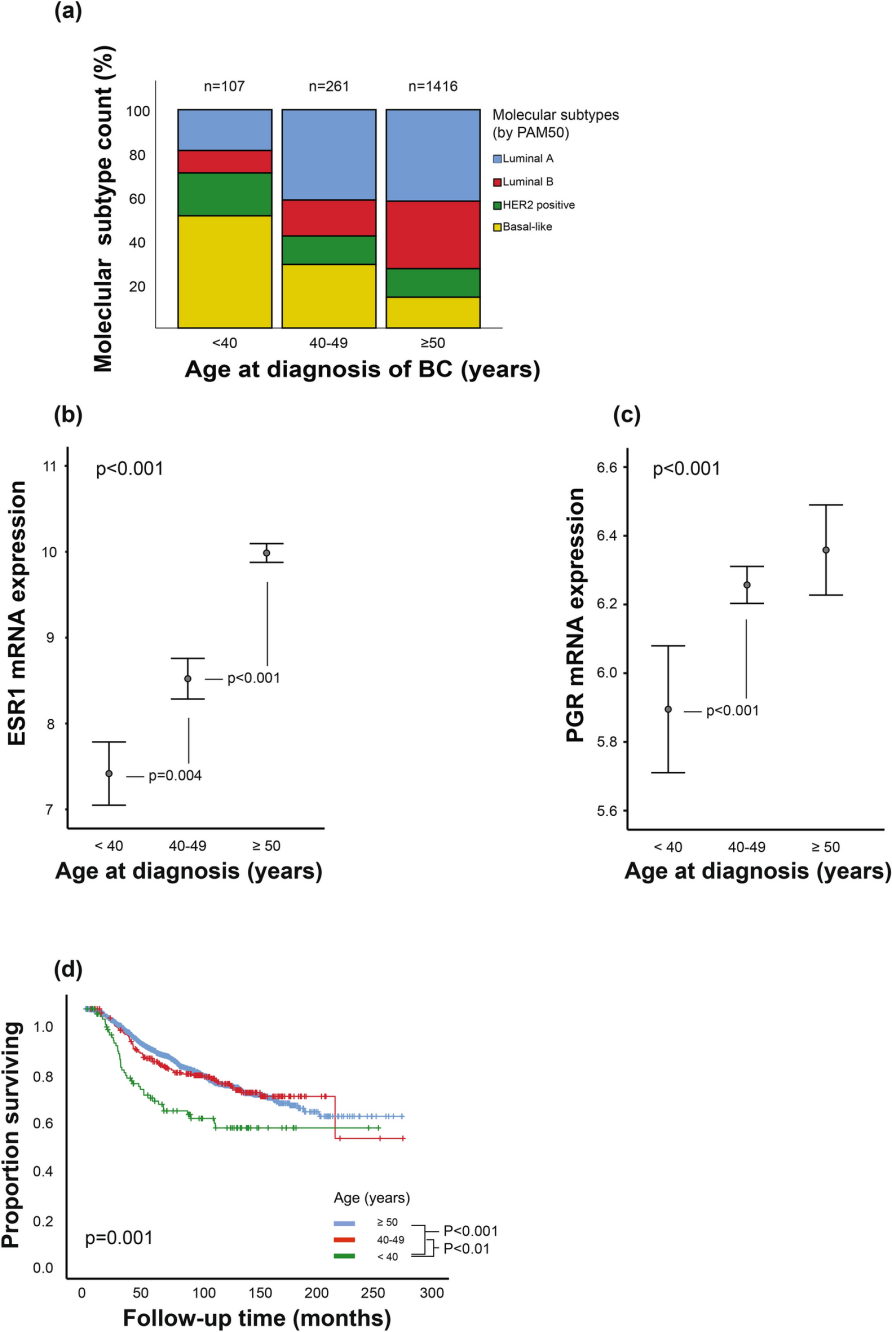
Age-related differences in molecular subtypes, hormone receptor expression and survival in breast cancer patients. (**a**) Distribution of molecular subtypes by percent of cases within each age groups. Molecular subtypes defined by PAM50. **(b-c)** *ESR1* (ER) and *PGR* (PR) mRNA expression across age groups. Data is presented by error-bars with 95% confidence interval of the mean, and p-values by the Kruskal–Wallis test. **(d)** Kaplan–Meier univariate breast cancer disease specific survival analysis according to age groups (log-rank test for difference). Age groups < 40 years; 40–49 years; ≥ 50 years, at time of diagnosis. Number of patients by age groups, n = < 40 years: 120; 40–49 years: 306; ≥ 50 years: 1566.

Young age was associated with more aggressive clinico-pathological tumor features including high histological grade, more frequent lymph node metastasis involvement and estrogen receptor alpha (ER) negativity (**Supplementary Table S1**). Patients below 40 displayed the most aggressive tumor features, followed by the patients aged 40–49 years of age, while those 50 years or older had the least aggressive tumor characteristics. A similar distribution in aggressiveness across the age groups was observed when investigating the PAM50 molecular subtypes, mRNA expression of estrogen- and progesterone receptors encoded by *ESR1* and *PGR*, and immunohistochemically evaluated ER protein-levels (Fig. 1a-c**; Supplementary Table S1**). Tumors of the youngest patient group displayed a higher proportion of molecular subtypes known to be associated with higher tumor aggressiveness, such as HER2-enriched and basal-like molecular subtypes, and lower ER protein and *ESR1*- and *PGR* mRNA gene expression levels, compared to older patients.

### Breast cancer patients below 40 years have poorer survival

Next, we performed survival analyses using the same age-groups as presented. In line with the more aggressive tumor characteristics, we demonstrated a significantly shorter overall survival for patients below 40 years of age (Fig. 1d). The two older age groups, aged 40–49 and over 50 years, had similar survival patterns and were not significantly different towards each other (10-year survival 73% vs 72%, p = 0.747). When stratified by molecular subtypes, patients below 40 years with tumors of the basal-like subtype demonstrated a trend of shorter disease-specific survival (**Supplementary Figure S1d**, 10-year survival 50% vs 67%, p = 0.062). No significant differences in age-dependent survival analyses were observed (**Supplementary Figure S1**).

### Young age of breast cancer patients is associated with increased stemness features

**Fig. 2:**
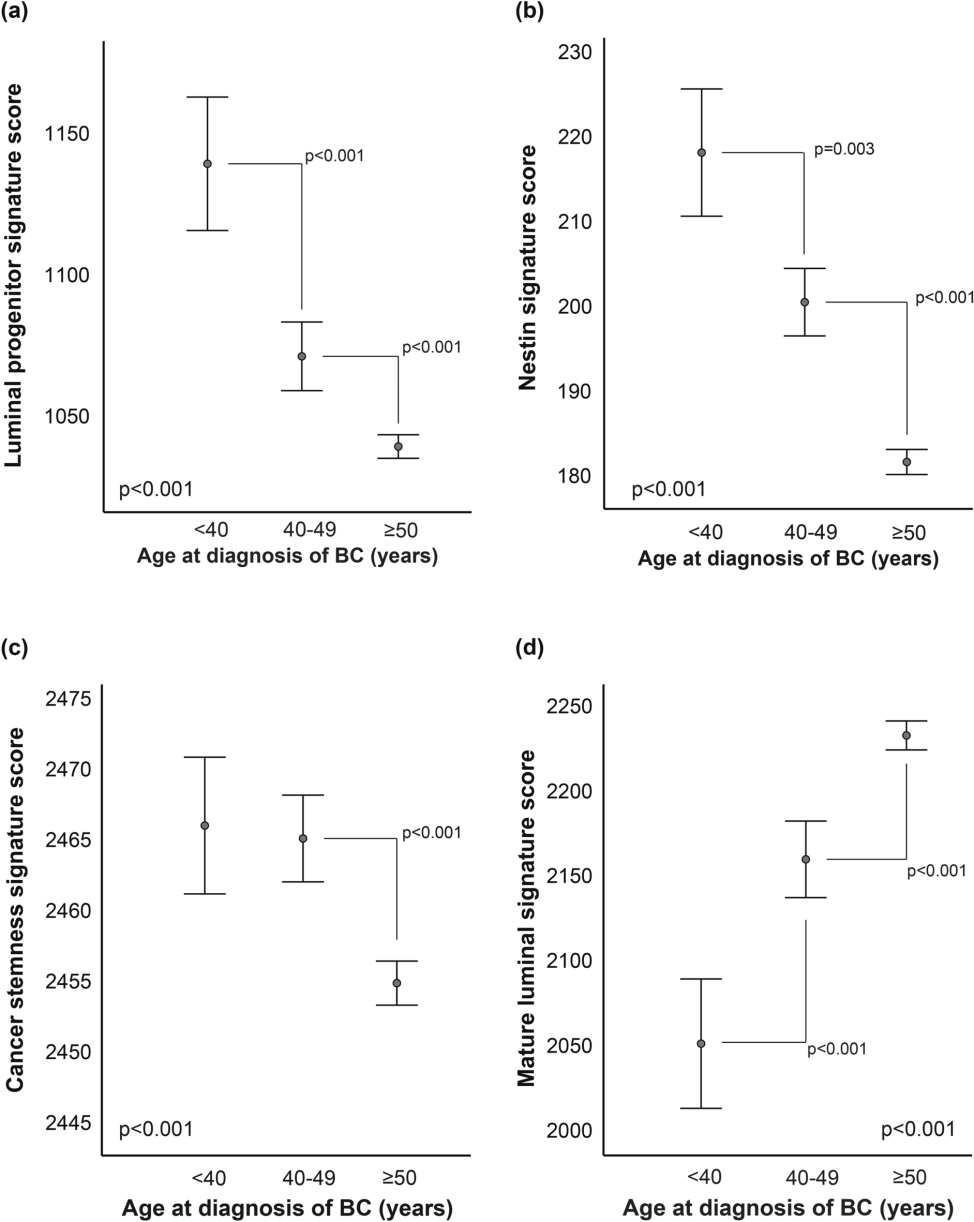
Gene expression signatures scores reflecting progenitor and stemness features across age groups. Higher scores of signatures reflecting **(a)** Luminal progenitor activation; **(b-c)** stemness features in breast cancer of the young. **(d)** Lower scores reflecting a mature luminal program in the young. Data is presented by error-bars with 95% confidence interval of the mean, and p-values by Kruskal–Wallis test.

To further explore the association between young age and the basal-like phenotype, we assessed how mRNA gene expression signatures of mammary stem cells and luminal progenitor cells were displayed in different age groups (Fig. 2). Age below 40 years was significantly associated with a high luminal progenitor signature score^16^and Nestin^17^ score reflecting cancer stemness (Fig. 2a-b, p < 0.001 and p = 0.003). A second score reflecting cancer stemness features^18^ displayed higher expression levels in the patient groups under 40 and 40–49 when compared to those over 50 (Fig. 2c, p < 0.001). Finally, a mature luminal signature score^16^ was expressed at lowest level in the youngest breast cancer patients compared to the two older age groups (Fig. 2d, p < 0.001).

The association between young age and higher cancer stem cell transcriptional patterns was also present within each of the PAM50-defined molecular subtypes (**Supplementary Figure S2)**. Patients below 40 presented with higher Nestin score in luminal A and basal-like subtypes compared to patients older than 50 years (**Supplementary Figure S2a**, p < 0.001 for both). Similarly, those below 40 also presented with higher cancer stemness score in luminal A subtype and higher progenitor score in basal-like subtype (**Supplementary Figure S2b-c**, p = 0.011 and p < 0.001). Even more differences were observed between patients aged 40–49 compared to the older patients (**Supplementary Figure S2a-c**), including Nestin score across all subtypes, supporting a link between breast cancer of the young, a basal-like phenotype, and tumors with progenitor and stem cell features.

### Younger breast cancer patients are more frequently treated with chemotherapy

**Fig. 3:**
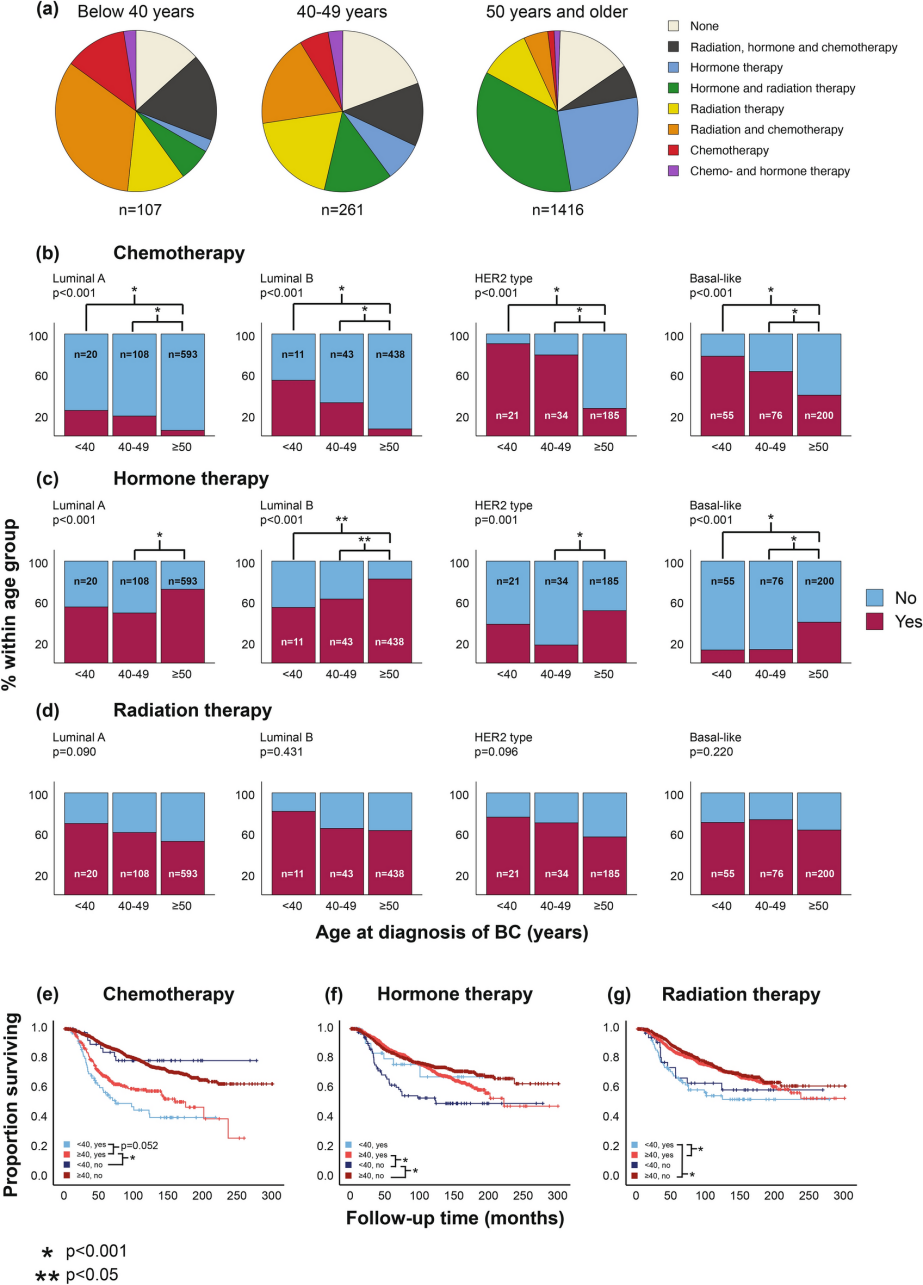
Age-related differences in treatment of breast cancer patients. **(a)** Distribution of cancer treatment received across age-groups. **(b-d**) Different treatments provided across age-groups, including chemotherapy (**b**), hormone therapy (**c**) and radiation therapy (**d**). P value by Pearson’s chi-squared test. **(e–g)** Breast cancer disease-specific survival according to age-groups at diagnosis and treatment received. All molecular subgroups are combined in plot. Kaplan–Meier univariate breast cancer specific survival (log-rank test for difference). Number of patients by age and treatment (yes/no) (**e**) Chemotherapy: n = Age < 40, yes: 78; Age < 40, no: Age 41; Age ≥ 40, yes: 343; Age ≥ 40, no: 1528. (**f**) Hormone therapy: n = Age < 40, yes: 35; Age < 40, no: 84; Age ≥ 40, yes: 1190; Age ≥ 40, no: 681. (**g**) Radiation therapy: n = Age < 40, yes: 82; Age < 40, no: 37; Age ≥ 40, yes: 1096; Age ≥ 40, no: 775.

Given the differences in molecular and clinical variables as well as survival, we next assessed if there were any age-related differences in single or combined treatment(s) (Fig. 3a**)**. Patients below 40 years more often received chemotherapy compared to patients older than 40 years (p < 0.001), also within luminal subtypes (p < 0.001) and across all molecular subtypes (Fig. 3b**)**. Patients under 40 years with luminal B and basal-like tumors were less frequently treated with hormone therapy (Fig. 3c). We did not observe any differences in radiation therapy across age-groups (Fig. 3d).

As young age is associated with tumor characteristics warranting chemotherapy, we next assessed the age-related differences in usage of chemotherapy in multiple logistic regression. The odds of receiving chemotherapy were 6.11 times higher for younger women after correcting for tumor size, lymph node involvement, tumor grade and molecular subtype by PAM50 (**Supplementary Table S2a**). The odds of receiving hormone therapy were 0.43 times lower for young women after correcting for ER-status, tumor size, lymph node involvement and tumor grade (**Supplementary Table S2b**). Young patients who underwent chemotherapy had the poorest survival prospects of all patient groups considering this treatment. The young patients who received chemotherapy presented shorter survival compared to older patients receiving chemotherapy (Fig. 3e; p = 0.052, 10-year survival 49% vs 58%). When considering hormone therapy, no differences were observed when comparing young and old patients with treatment (Fig. 3f). Young patients who received radiation therapy had the poorest survival (Fig. 3g**, 10**-year survival 52%) compared to older with or without radiation therapy (10-year survival; 72% and 73%, both p < 0.001). For both hormone- and radiation therapy, no significant differences were observed when comparing young patients receiving therapy to those without.

## Discussion

Our description of more aggressive tumor characteristics seen in young women with breast cancer corresponds well with current literature^7,8^. As a result, young women showed poorer survival compared to older patients, with a trend of shorter survival within the basal-like subgroup. Previous studies on breast cancer of the young have demonstrated similar relations between young age and survival in different tumor subtypes^4^. Similar to our recent study, using in-house data^8^, our present findings, based on external data, also show differences among the patients below 40 and 40–49 years of age. This underscores a likely unique age-related biology among young breast cancer patients.

Our results support a link between breast cancer of the young, a basal-like phenotype, and tumors with increased progenitor and stem cell features. In previous studies, mammary stem cells have been proposed to have important roles in breast cancer development and progression^19^, and have therefore been suggested as potential relevant targets in new treatment strategies^19,20^. It has been proposed that age-related accumulating genetic- and epigenetic alterations stimulate cancer stem cell development^21^. In contrast, our data indicates more frequent cancer stemness properties in the younger age groups – providing additional perspectives on age and cancer stem cells that need further investigation.

The association between germline *BRCA1* and *BRCA2* mutations and young age could potentially explain parts of the high rate of the basal-like subtype we observed. Unfortunately, we lack information regarding germline *BRCA1/2* mutations, as this information is considered patient sensitive data and therefore not released. However, from the literature, we can expect around 9% of patients under the age of 41 years to be carriers of *BRCA1/2*germline mutations^22^, compared to 3% for the general breast cancer population^23^. The *BRCA2* mutation carriers are more likely to develop hormone receptor-positive tumors, while about 60–70% of the *BRCA1*mutation carriers will advance into the triple-negative BC subtype^24^. Both *BRCA1* and *−2* carriers are more likely to develop early-onset breast cancer, but the association is stronger for *BRCA1*mutated patients^25^. In summary, after correcting for *BRCA2* mutations, *BRCA1* mutations in older age groups, and the proportion of basal-like tumors in *BRCA1* mutated patients, only a smaller fraction of the basal-like tumors in young can be directly attributed to *BRCA1* germline mutations.

Current management of breast cancer relies on molecular subgroup stratification and other biomarkers to tailor treatment. However, given the age-dependent differences in biology and survival outcome, an additional focus on adjusting treatment according to age is needed. Here, we show that young women below 40 years of age more often receive chemotherapy and less often hormone therapy when adjusted for tumor size, histologic grade, lymph node status, and molecular subtypes or ER status, and when assessed within each of the individual molecular subgroups. There might be several factors underlying the observation that young patients more often receive chemotherapy treatment. Young women are more often presented with aggressive tumor features, which may guide clinicians to choose chemotherapy as the preferred treatment strategy. Younger women are also at risk of being diagnosed at later stages, as breast cancer screening for those below 50 generally is lacking. However, when adjusting for clinico-pathologic variables in multivariable analyses, we found that young women were still more likely to receive chemotherapy, as described by Murphy et al*.*^26^. Our observation was further supported by a similar trend in the patient group below 50 years of age in our data (p = 0.092). Taken together, this suggests that young women are more often treated with chemotherapy, more than what can be explained by aggressive tumor features alone, and that these age-dependent discrepancies should be considered when allocating patients to this treatment.

We used PAM50 to categorize molecular subgroups since the immunohistochemistry information, generally used for clinical guidance, was incomplete in our data. It is worth noting that some ER negative and positive cases may fall into the PAM50 luminal and non-lumina subgroups, respectively, although the majority of cases substantially overlap between these two classifications. A few of the patients with luminal tumors who did not receive hormone therapy, and patients with non-luminal tumors receiving hormone therapy, may be due this. Despite this, young age remains associated with receiving less hormone therapy even after correcting for ER status and other clinico-pathologic tumor features.

Interestingly, when investigating the relation of age and treatment combined in survival analyses, we found shorter survival in the young patients who received chemotherapy compared to older patients. Studies on breast cancer have shown increased resistance to chemotherapy in cancer stem cells^10,20^. Given the increased stemness features also found in breast cancer of the young by Han et al*.*^11^*,* supporting the results from our present study, we speculate that age-related cancer stemness differences may contribute to shorter survival in young women, also in patient groups receiving treatment. No clear survival differences were found when combining age with hormone- or radiation therapy.

There may be factors that influence the interpretation of our results. Premenopausal women have lower compliance to hormone therapy due to increased adverse effects^27^and are in general more motivated for chemotherapy^28^. Somatic alterations that may promote hormone resistance, such as *GATA3 *mutations, found more frequently in younger women^29^, potentially contributing to different effects of hormonal therapy by age groups. Another consideration is that hormone-sensitive tumors may eventually become resistant to endocrine therapy in metastatic settings by *ESR1 *(ER-gene) mutations (e.g. hotspots p.Tyr537Ser, p.Tyr537Asn, and p.Asp538Gly) after long-term anti-estrogen treatment^30^.

The public METABRIC dataset also has some limitations. Given that only 6% of the patients in the datasets were diagnosed below 40 years of age, and that the majority of these (70.8%) are classified as non-luminal molecular types (HER2 and basal-like), the small numbers may influence statistical power in our analysis. Also, for some of the clinical variables such as staging and details of treatment, we lack complete information. Finally, some variables are unavailable, which we believe would have been useful in the context of our study, in particular the menopause status.

## Conclusions

Our results confirm that young women with breast cancer face a more challenging disease due to more aggressive tumor characteristics, more frequent basal-like subtype, and increased associations with stemness features. We demonstrate reduced survival for the younger age group despite more abundant use of chemotherapy. Given the age-related differences in tumor biology and treatment, we believe that a specific focus on young women is warranted in future research and clinical trials, to improve treatment, reduce overtreatment, and increase survival for this patient group.

## Supplementary Information

Supplementary Information.

## Data Availability

Data from the Molecular Taxonomy of Breast Cancer International Consortium (METABRIC) is available at https://ega-archive.org/studies/EGAS00000000083.

## Abbreviations

ER: Estrogen receptor
METABRIC: Molecular Taxonomy of Breast Cancer International Consortium
