# Supplementary material for: Distinct clinicopathological features and treatment differences in breast cancer patients of young age
Source: Sci Rep. 2025 Feb 15;15:5655. doi: 10.1038/s41598-025-90053-9 (PMC11830014; doi:10.1038/s41598-025-90053-9)
Supplement: Supplementary file 1 — Supplementary Information. [file 41598_2025_90053_MOESM1_ESM.pdf]

Supplementary Table S1. Clinico-pathologic features across age groups

	Age at diagnosis (years)			
	<40	40-49	≥50	
	n (%)	n (%)	n (%)	P
**Histological grade**				
1	1 (0.9)	25 (9.8)	120 (8.9)	<0.001
2	20 (18.7)	85 (33.2)	568 (42.1)	
3	86 (80.4)	146 (57.0)	660 (49.0)	
**Tumor diameter**				
≤2.0 cm	33 (31.4)	91 (35.4)	414 (29.4)	0.16
>2.0 cm	72 (68.6)	166 (64.6)	992 (70.6)	
**Nodal status**				
Negative	36 (33.6)	136 (52.1)	750 (53.0)	0.001
Positive	71 (66.4)	125 (47.9)	666 (47.0)	
**ER (IHC)**				
Positive	35 (33.0)	158 (61.2)	1149 (83.0)	<0.001
Negative	71 (67.0)	100 (38.8)	235 (17.0)	
**Molecular subtype (PAM50)**				
Luminal A	20 (18.7)	108 (41.4)	593 (41.9)	<0.001
Luminal B	11 (10.3)	43 (16.5)	438 (30.9)	
HER2 type	21 (19.6)	34 (13.0)	185 (13.1)	
Basal-like	55 (51.4)	76 (29.1)	200 (14.1)	

n: number of patients; P: p-values. P-values by Pearson's chi-square test. Missing data: Histologic type: n=2; histologic grade: n=73; tumor diameter: n=16; ER: n=36.

Supplementary Figure S1.

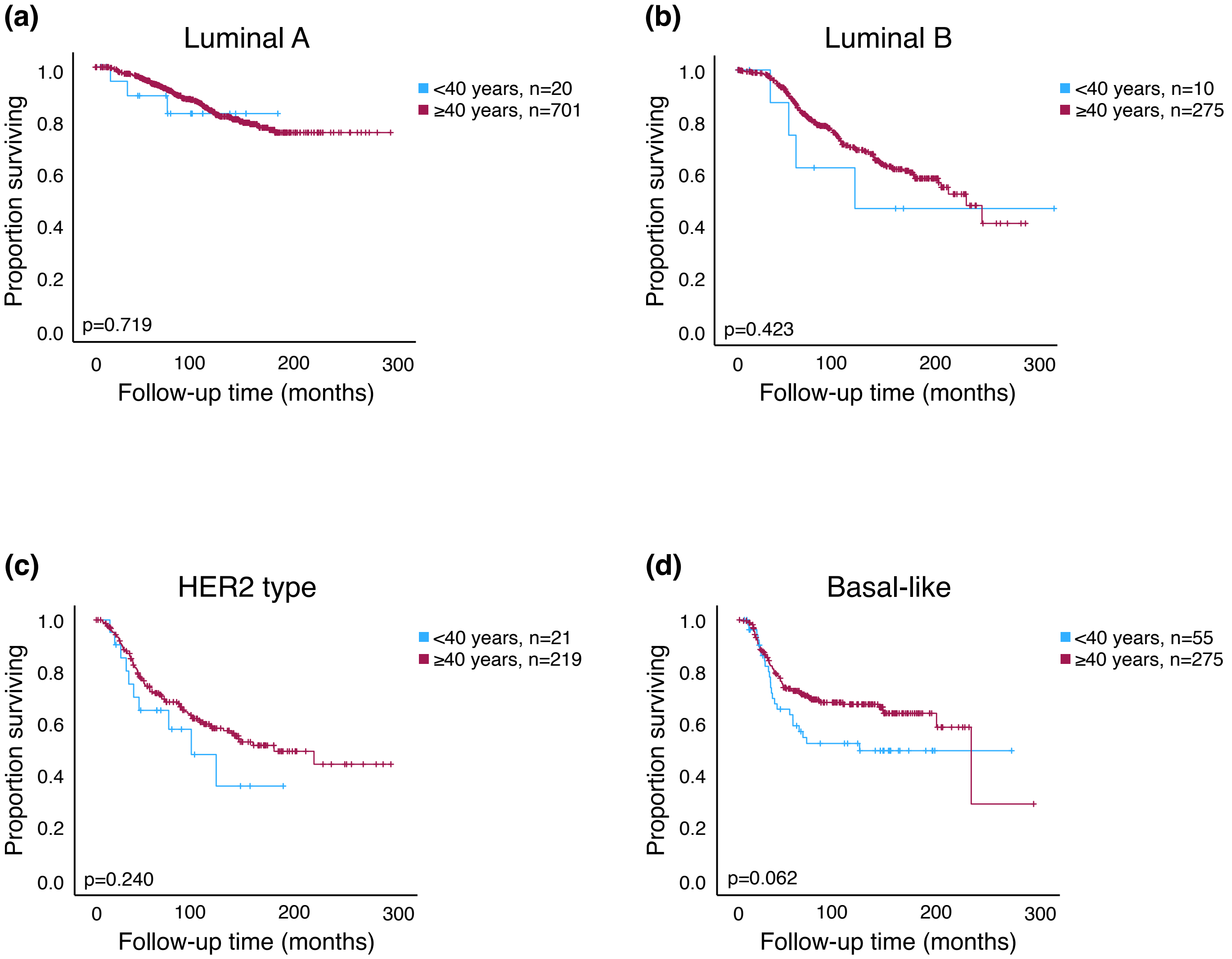

Supplementary Figure S1. Kaplan-Meier breast cancer-specific survival analysis, split by molecular subgroup and across age groups. Kaplan-Meier univariate breast cancer disease specific survival analysis according to age groups under 40 years vs 40 years and older (log-rank test for difference). Split by molecular subgroups.

Supplementary Figure S2.

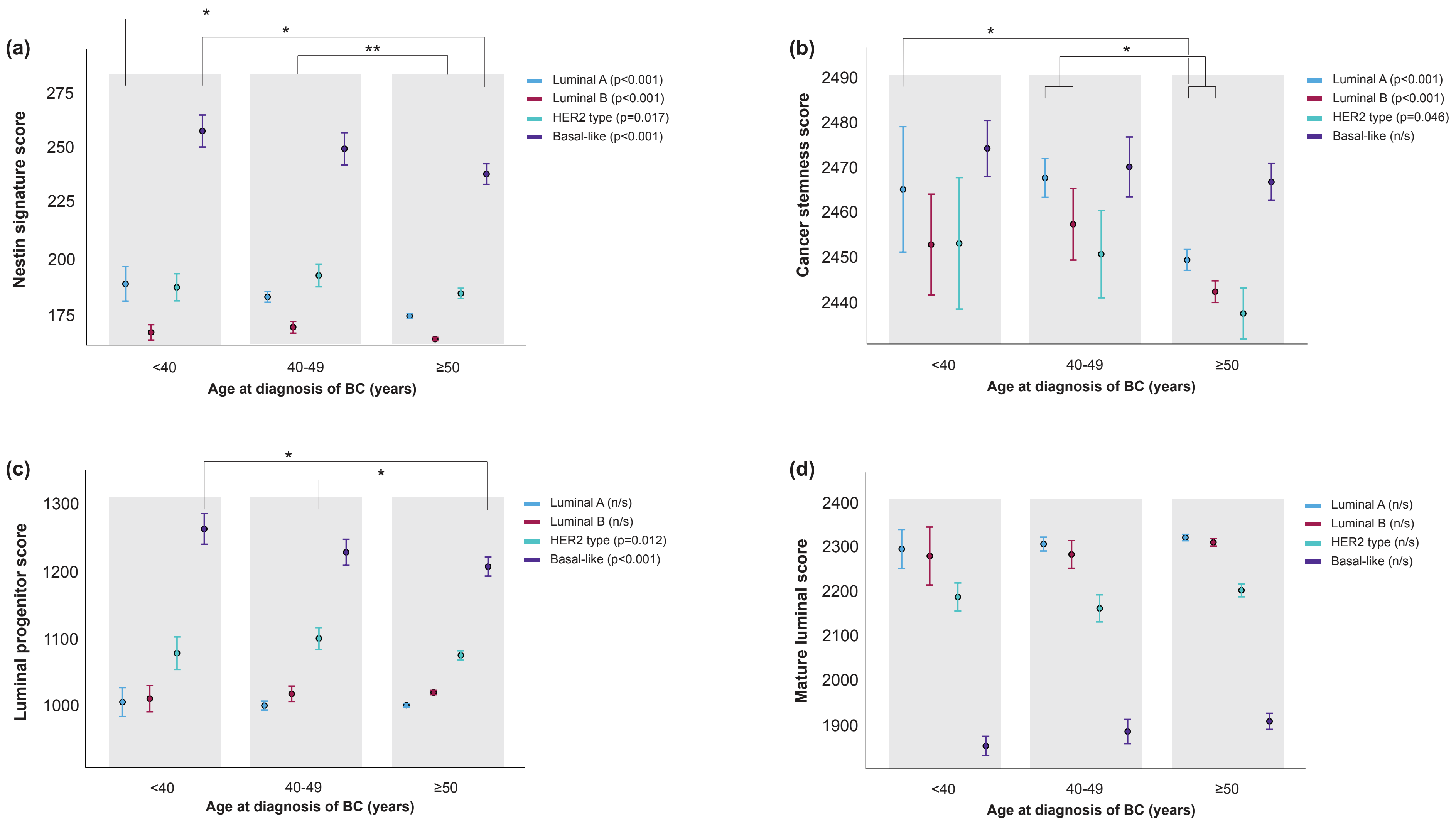

* p<0.05

** all subgroups are significantly (p<0.05) different expressed

Supplementary Figure S2. Gene expression signatures reflecting progenitor and stemness features across age groups (METABRIC combined cohorts n=1992). Higher scores of the signatures reflecting A-B) stemness features in breast cancer of the young; D) Luminal progenitor activation. C) Lower scores of the signature reflecting a mature luminal program in the young. Data is presented by error-bars with 95% confidence interval of the mean, p-values comparing several groups by the Kruskal-Wallis test and p-values for direct comparison by Mann-Whitney U test.

Supplementary Table S2. Prediction of receiving chemotherapy and hormone therapy (yes/no) by multivariable logistic regression

(a) Chemotherapy

Variable	n	(%)	OR	95% CI	P
**Age (years)**					
≥40	1593	(93.8)	1		<0.001
<40	105	(6.2)	6.11	(3.42-10.90)	
**Tumor size**					
≤2.0 cm	728	(42.9)	1		0.038
>2.0 cm	970	(57.1)	1.42	(1.02-1.97)	
**Nodal status**					
Negative	867	(51.1)	1		<0.001
Positive	831	(48.9)	12.47	(8.57-18.14)	
**Histological grade**					
1 and 2	812	(47.8)	1		<0.001
3	886	(52.2)	2.59	(1.82-3.68)	
**Luminal vs non-luminal**					
Luminal	1156	(68.1)	1		
Non-luminal	542	(31.9)	7.96	(5.72-11.07)	<0.001

(b) Hormone therapy

Variable	n	(%)	OR	95% CI	p Value
**Age**					
≥40	1573	(93.8)	1		0.001
<40	104	(6.2)	0.43	(0.25-0.72)	
**Tumor size**					
≤2.0 cm	714	(42.6)	1		<0.001
>2.0 cm	963	(57.4)	1.76	(1.37-2.26)	
**Nodal status**					
Negative	848	(50.6)	1		<0.001
Positive	829	(49.4)	6.21	(4.64-8.33)	
**Histological grade**					
1 and 2	797	(47.5)	1		<0.001
3	880	(52.5)	1.76	(1.33-2.33)	
**ER status**					
Negative	395	(23.6)	1		<0.001
Positive	1282	(76.4)	25.36	(17.44-36.86)	

n: number of patients; OR: odds ratio; CI: confidence interval; P: p-values; ER: estrogen receptor. All variables are categorical; Luminal includes PAM50 luminal A and B, non-luminal PAM50 HER2 and basal-like; ER status by immunohistochemistry. PAM50 normal like subgroup was excluded from the analysis. Missing cases (A) n=86 (B) n=107.
